# Estimation and influence of blood loss under endoscope for percutaneous endoscopic lumbar discectomy (PELD): a clinical observational study combined with in vitro experiment

**DOI:** 10.1186/s13018-020-01797-1

**Published:** 2020-07-25

**Authors:** Dong Dong Sun, Dan Lv, Wei Zhou Wu, He Fei Ren, Bu He Bao, Qun Liu, Ming Lin Sun

**Affiliations:** 1 Department of Orthopedic, Characteristic Medical Center of Chinese People’s Armed Police Force, 220 Cheng Lin Road, He Dong District, Tianjin, 300171 China; 2 Logistics University of Chinese People’s Armed Police, Tianjin, 300300 China; 3 Department of Neurology, The 985th Hospital of the Joint Logistics Support Force of the PLA, Taiyuan, 030001 China; 4 Clinical laboratory, Characteristic Medical Center of Chinese People’s Armed Police Force, Tianjin, 300171 China

**Keywords:** Lumbar disk herniation, Percutaneous endoscopic lumbar discectomy, Intraoperative blood loss, Endoscopic hemostasis, Pain management

## Abstract

**Purpose:**

The purpose of this study is to come up with new methods to quantitate the blood loss under endoscope and explore the influence of blood loss on percutaneous endoscopic lumbar discectomy (PELD).

**Methods:**

Clinical research and in vitro experiment are combined. In the in vitro experiment, 2.0-ml blood was diluted in different ratio to simulate the rinse solution of PELD, the hematocrit method (HCT-M) and red blood cell count method (RBC-M) were came up to estimate blood loss and the new methods were calibrated with the direct measurement method (Direct-M). In clinical research, 74 patients with L5/S1 disk herniation were treated with PELD, and HCT-M and the empirical method (EMP-M) were used to estimate the blood loss under endoscope. According to blood loss, all patients were divided into group A (≤ 10 ml) and group B (> 10 ml). The blood loss, operation time, fluoroscopy frequency, visual analog scale (VAS), and Oswestry Disability Index (ODI) scores were compared between the two groups.

**Results:**

In the in vitro experiment, the hematocrit of the rinse solution was always stable over time. The estimated blood loss by HCT-M was stable and quite approximate to actual blood volume (2.0 ml) whatever the blood dilution ratio, while according to RBC-M, the estimated blood loss was close to the actual blood volume only when the dilution ratio was greater than 300 times. In clinical research, the blood loss estimated by HCT-M was higher than that by EMP-M in both groups (*P* < 0.05). There was a significant difference between group A and group B in blood loss (7.40 ± 1.61 vs 19.91 ± 10.94 ml), operation time (80.51 ± 34.70 vs 136.51 ± 41.88 min), and fluoroscopy frequency (6.92 ± 1.52 vs 11.11 ± 2.32 times) (*P* < 0.05). The VAS and ODI scores in group B were higher than that in group A 1 week after operation (*P* < 0.05); however, the scores were not different between the two groups at pre-operation (*P* > 0.05).

**Conclusion:**

HCT-M is a reliable method to estimate endoscopic blood loss in PELD. The amount of endoscopic blood loss affects the operative procedure in operation time and fluoroscopy frequency, as well as clinical effects in VAS and ODI scores after operation in short term.

## Introduction

Lumbar disk herniation (LDH) is a very common disease in clinical which is associated with both genetic and environmental factors. The treatment for LDH includes conservative therapy and surgery and the standard open discectomy has largely been replaced by microdiscectomy [1]. According to a Norway longitudinal observation study of 34,639 surgical cases of lumbar disk herniation, microdiscectomy was specified in 23,929 patients (69%) [2]. Percutaneous endoscopic lumbar discectomy (PELD) is one of the microdiscectomy methods which has made significant progress and is well accepted by surgeons in recent years with the development of endoscopic technique and surgical instruments [3, 4]. Compared with other surgical methods, this technique has less trauma, fewer blood loss, faster postoperative recovery, higher cost-effectiveness, and shorter postoperative bed time and hospitalization time [5–7]. Thus, PELD has become one of the mainstream operative methods for the treatment of LDH [8]. According to the approach, the percutaneous endoscopic lumbar discectomy is divided into the posterior approach and the lateral posterior approach [9, 10], whereas the lateral posterior approach is divided into the Yeung endoscopic spine system (YESS) [11] and the transforaminal endoscopic spine system (TESSYS) [12, 13]. Depending on the segment of disk herniation, the type of disk herniation, and individual surgical habits, the surgeon may perform the endoscopic surgery with different approaches [9, 14–16].

No matter which approach, a safe working zone is commonly required to achieve the percutaneous endoscopic access into the lumbar areas. The safe working zone should include the following characteristics: clear view, direct access, selective discectomy, and feasibility of local anesthesia [3]. Among them, the clear view under the endoscope is the most important characteristic, and intraoperative bleeding is the main factor affecting it. Massive endoscopic bleeding can seriously affect the operation and even terminate the operation, potentially causing complications such as dural sac tears, epidural hematoma, nerve root injury, incomplete removal, and infection [17, 18]. However, there is no study on the assessment and countermeasures of endoscopic blood loss of PELD until now. Endoscopic blood loss means the blood loss under the endoscope, which does not include the blood loss during puncture.

**Fig. 1:**
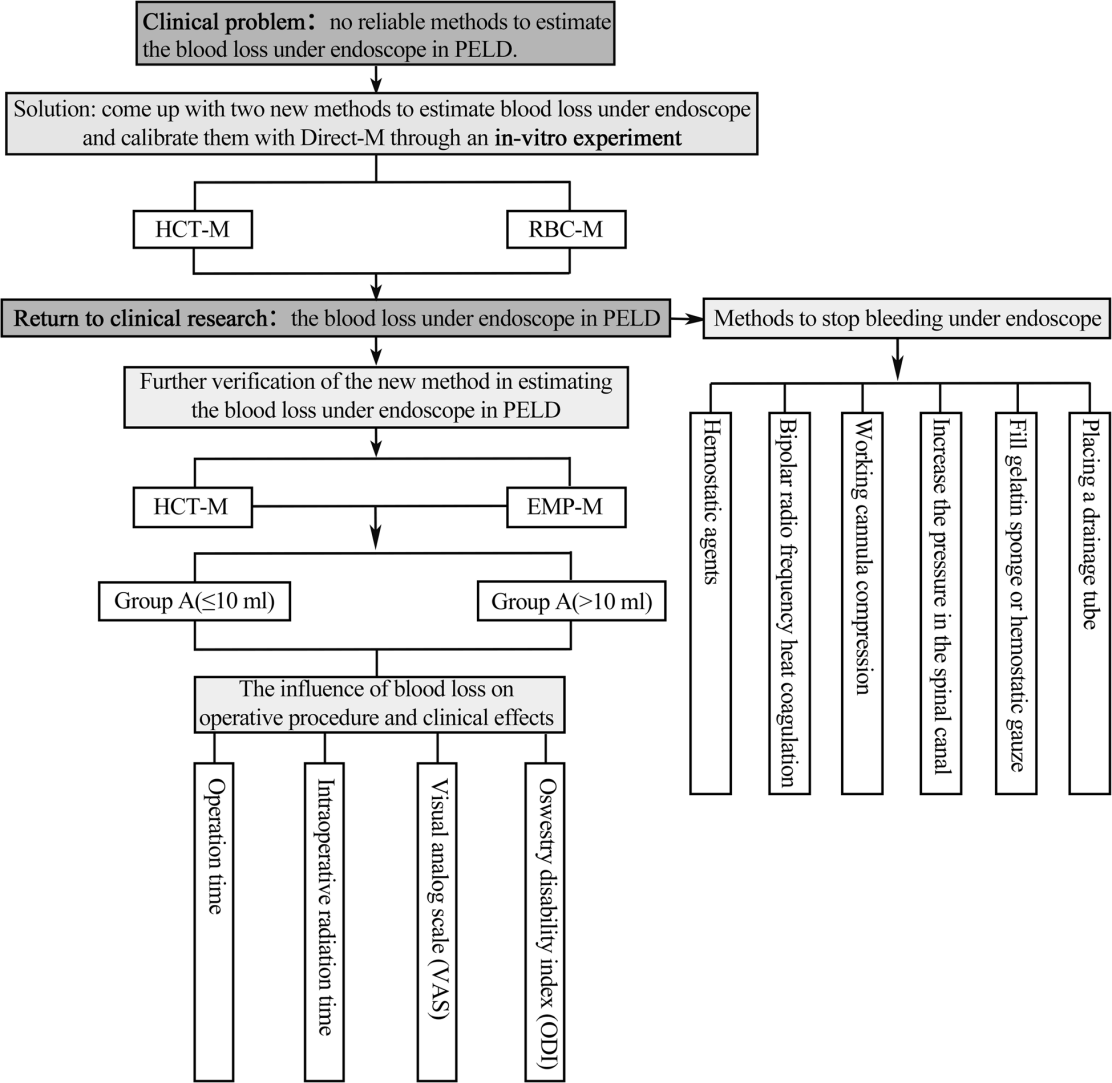
Graphic abstract (PELD, percutaneous endoscopic lumbar discectomy; HCT-M, hematocrit method; RBC-M, red blood cell count method; Direct-M, direct measurement method; EMP-M, empirical method)

The goal of this study was to find new methods to estimate the blood loss under the endoscope, explore the influence of blood loss on operative time, intraoperative radiation time, Oswestry Disability Index (ODI), and visual analog scale (VAS) scores, analyze the cause of intraoperative bleeding, and summarized the hemostasis systematically. We clarify the patient selection for the percutaneous endoscopic transforaminal discectomy approach for L5/S1 disk herniation (Fig. 1).

## Materials and methods

### In vitro experiment

#### Experimental principle and purpose

Hematocrit is one of the indicators in routine blood examination. RBCs can maintain their normal morphology in 0.9% saline for a long time; thus, the amount of blood loss under endoscope can be estimated by detecting the hematocrit and RBC count of the rinse solution [19]. The new methods were tested and calibrated by comparing them with the direct measurement method (Direct-M) through the in vitro experiment.

#### Materials and animals

Japanese white rabbits were bought from Chauncey biology (SCXK 2016-0007, Beijing, China), Sysmex XN-1000 automatic modular animal blood fluid analyzer (Japan, Sysmex Corporation), and Sysmex UF-1000i automated urine analyzer (Japan, Sysmex Corporation).

#### Experiment procedure (Fig. 2)

Four Japanese white rabbits were enrolled into the experiment. Firstly, 4 ml of blood was drawn from each rabbit, and 2 ml of the sample was used to make routine blood examination for determining the hematocrit and RBC count of normal rabbit blood (Sysmex XN-1000, Japan). The other half of the blood was measured directly by a syringe first (Direct-M), then it was injected into 100 ml of 0.9% saline, and further diluted 100 times, 200 times, 300 times, 400 times, and 800 times, to simulate the rinse solution of PELD. Next, the hematocrit and RBC count of simulative rinse solution were detected respectively (Sysmex UF-1000i, Japan). Finally, the endoscopic blood loss was calculated according to the following formulas.

**Fig. 2:**
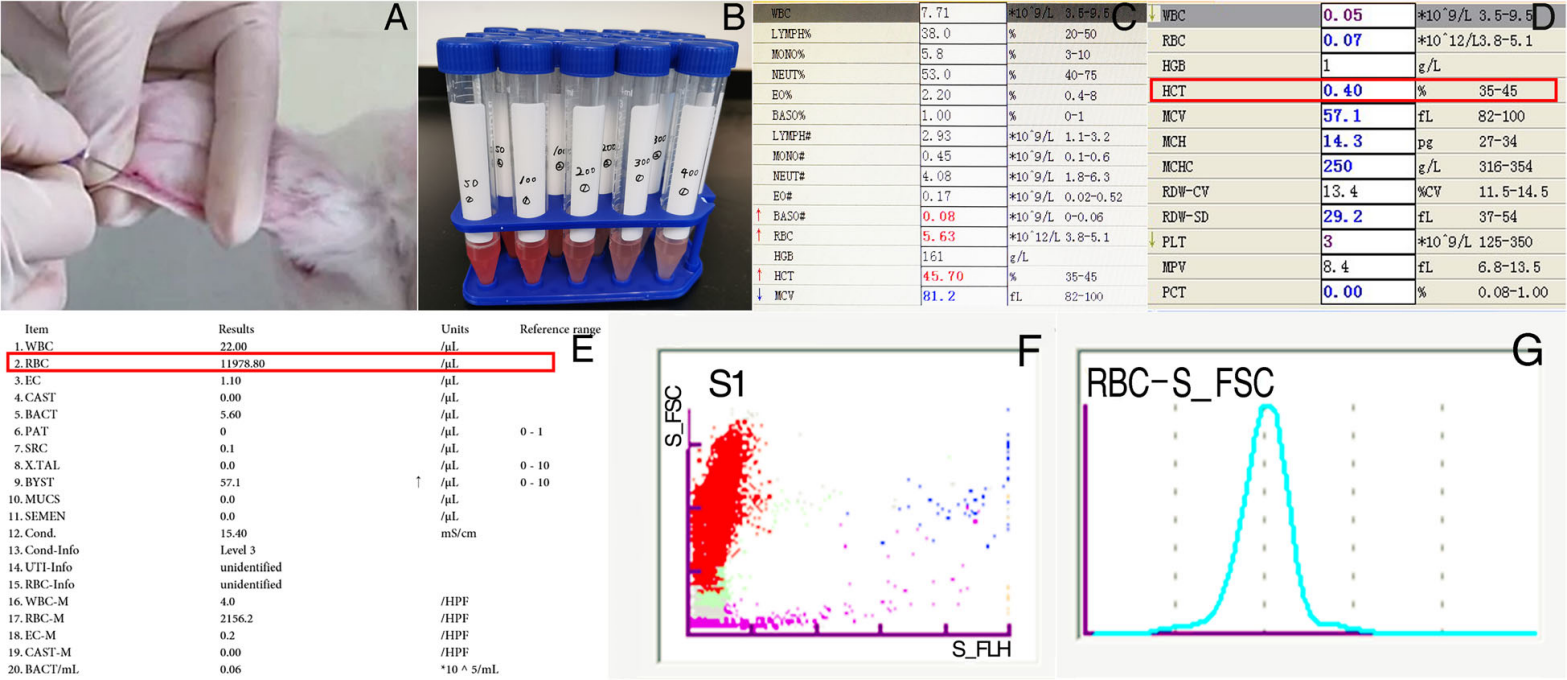
In vitro experiment. **a** Drawing blood from Japanese white rabbits. **b** Simulated rinse solution with different dilution times. **c** Normal routine blood examination of rabbit. **d** The hematocrit of simulated rinse solution. **e** The RBCs’ counts were examined by Sysmex UF-1000i automated urine analyzer. **f**, **g** Fluorescent flow cytometry was used to count RBCs in the simulated rinse solution

Hematocrit method (HCT-M): *V*_blood_ =$\frac{Hct1}{Hct2}$_×_*V*_rinse solution_

RBC count method (RBC-M): *V*_blood_ =$\frac{RBC1}{RBC2}$_×_*V*_rinse solution_

Direct measurement method (Direct-M): *V*_blood_ = 2 ml

*V*_blood_: the volume of blood loss under endoscope

*V*_rinse solution_: total volume of rinse solution

Hct_1_: the hematocrit of rinse solution

Hct_2_: the hematocrit of normal blood

RBC_1_: the RBC count of rinse solution

RBC_2_: the RBC count of normal blood

### Clinical research

#### General information

In this observational study, 74 patients who underwent PELD via the transforaminal approach at L5-S1 in our department between September 2017 and September 2019 were included. According to the endoscopic blood loss (HCT-M), all patients were divided into two groups: group A (≤ 10 ml) and group B (> 10 ml). There was no statistical difference in sex, age, disk herniation types, and symptoms/signs between group A and group B (Table 1).

**Table 1:**
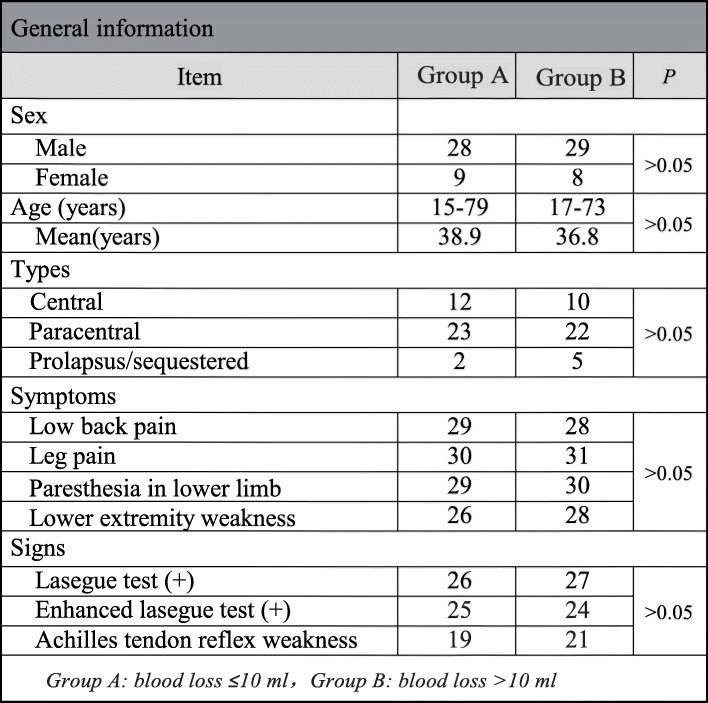
General information

Group A, endoscopic blood loss ≤ 10ml; group B, endoscopic blood loss > 10ml

The patient inclusion criteria were as follows: (1) typical symptoms and sign: low back pain, leg pain, accompanied by radiating pain in the lower limb with a typical sign, such as a positive Lasegue sign; (2) imaging examination: lumbar radiograph, computed tomography (CT), and/or magnetic resonance imaging (MRI) examination indicating a herniated disk at L5-S1; (3) no response to 6 weeks of conservative therapy, such as non-steroid anti-inflammatory drug, physical therapy, and bed rest; and (4) operative approach: transforaminal approach.

The exclusion criteria were as follows: extreme lateral LDH; lumbar inflammation, tuberculosis, or tumor; multiple segments of disk herniation; and lumbar spondylolisthesis, lumbar stenosis, and a surgical history involving the corresponding segment.

### Surgery procedure and methods to estimate blood loss

All the operations were performed by the same experienced surgeon; the surgery procedure was the same as previously reported [20]. Before the operation, the whole negative pressure suction system was pre-flushed with heparin saline. During surgery, a continuous flow of irrigation fluid (0.9% saline) was used to prevent any clotting and residual blood in the suction system and the rinse solution was collected carefully without any loss. After surgery, the blood loss under endoscope was estimated according to the doctor’s personal experience (empirical method, EMP-M) or the HCT-M. The steps of the HCT-M are as follows: (1) make a routine blood examination before the operation (Sysmex XN-1000, Japan). (2) Collect the rinse solution of PELD, remove the tissue from the rinse solution with a proper filter (Cell Trics, 04-0042-2314, 10 μm, Sysmex Corporation, Japan), and measure the volume of the solution. (3) Mix the rinse solution adequately and detect the hematocrit of the rinse solution (Sysmex XN-1000, Japan). (4) Calculate the endoscopic blood loss according to the formula mentioned previously.

#### Effectiveness evaluation

All patients were followed up for 1 week. The endoscopic blood loss, operation time, fluoroscopy frequency, and surgical curative effect were compared between group A and group B. The clinical effectiveness was evaluated 1 week after the operation according to the ODI and VAS.

### Statistical analysis

All data were analyzed with the SPSS software (version 25.0, SPSS Inc., Chicago, IL) for Windows. Quantitative data are expressed as $\overline{X}$ ± s, and the independent sample *t* test was used for comparisons between the two groups. Qualitative data are expressed as count and percentage and were evaluated by the chi-square test. The result was considered statistically significant if *P* < 0.05.

## Results

### In vitro experiment

**Fig. 3:**
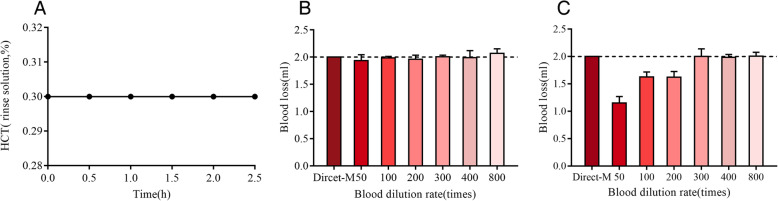
The results of in vitro experiment. **a** The hematocrit of the rinse solution was stable over time (diluted 100 times). **b** HCT-M, the calculated blood loss was approximate to the Direct-M (2.0 ml) whatever the blood dilution ratio. **c** RBC-M, the calculated blood loss is close to the Direct-M only when the blood dilution ratio was greater than 300 times

The hematocrit of the rinse solution was always stable over time, for example, when one sample of the rabbit blood was diluted 100 times, the hematocrit of the rinse solution was 0.3% all the time (Fig. 3a). In HCT-M, the calculated blood loss was stable and quite approximate to the Direct-M (2.0 ml) whatever the blood dilution ratio (Fig. 3b). In RBC-M, the calculated blood loss was lower than the Direct-M; however, when the blood dilution ratio was greater than 300 times, it was very close to the Direct-M (Fig. 3c). In summary, HCT-M is more stable and reliable in evaluating endoscopic blood loss when compared with RBC-M.

### Clinical research

#### Methods to estimate endoscopic blood loss

Two different methods were used to estimate endoscopic blood loss. The blood loss of all patients varies from 4.25 to 151.37 ml. In group B, two patients’ endoscopic blood loss was massive, and the largest amount volume of blood loss reached 151.37 ml according to HCT-M. One patients’ bleeding was stopped quickly with bipolar RF heat coagulation and gelatin sponge stuffing; the other patient had to change to open surgery. Both in group A and group B, there was a significant difference between HCT-M and EMP-M in evaluating endoscopic blood loss (*P* < 0.05). The detailed results are provided in Table 2.

**Table 2:**
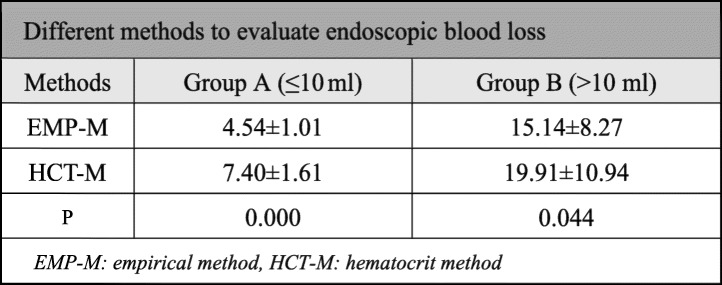
Different methods to evaluate endoscopic blood loss

*EMP-M* empirical method, *HCT-M* hematocrit method

#### Clinical effects comparison

In group A, the endoscopic blood loss was 7.40 ± 1.61 ml, operation time was 80.51 ± 34.70 min, and fluoroscopy frequency was 6.92 ± 1.52 times. In group B, the endoscopic blood loss was 19.91 ± 10.94 ml, operation time was 136.51 ± 41.88 min, and fluoroscopy frequency was 11.11 ± 2.32 times. There was a significant difference between group A and group B in endoscopic blood loss, operation time, and fluoroscopy frequency (*P* < 0.05). The VAS and ODI were used to estimate postoperative effects, and the results indicated that there was a significant improvement in leg and waist pain in both groups when compared with pre-operation scores (*P* < 0.05). There was no significant difference in VAS and ODI scoring between the two groups at pre-operation (*P* > 0.05). However, the VAS and ODI scores in group B were higher than those in group A 1 week after the operation (*P* < 0.05) (Table 3).

**Table 3:**
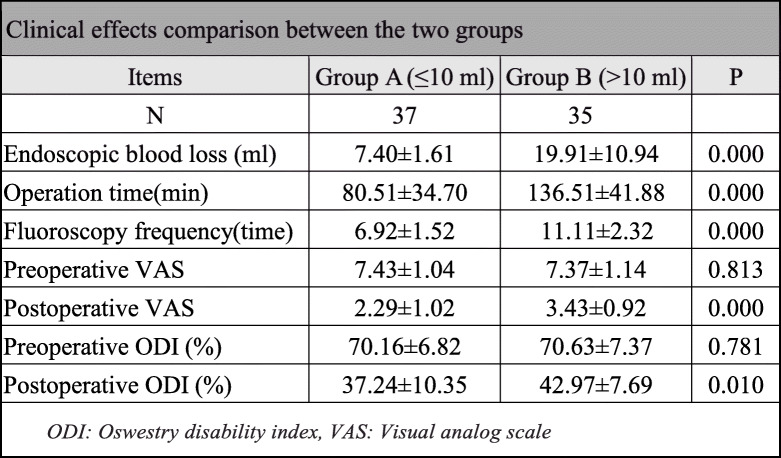
Clinical effects’ comparison between the two groups

*ODI* Oswestry Disability Index, *VAS* visual analog scale

## Discussion

Bleeding is one of the most common surgical complications of herniated lumbar disk surgeries [2]. The blood loss under the endoscope during PELD is generally less than 10 ml, but there also can be massive bleeding. A large amount of bleeding may result in an unclear view, lengthen the time of operation, increase the frequency of radiographic examination, and make postoperative VAS and ODI scores higher. Thus, evaluating the endoscopic blood loss accurately is very important to evaluate the curative effects of surgery, as well as for clinical studies. Currently, the empirical approach is the most popular approach used in clinical practice. However, the approach is convenient but not accurate. Estimation of the endoscopic blood loss in the same operation can vary widely from surgeon to surgeon. The HCT-M and RBC-M are two new methods we devised to estimate endoscopic blood loss in PELD.

The clinical study showed that the volume of 0.9% saline used for flushing tissues varied from 2.00 to 8.00 l and the diluted times of rinse solution varied from 126 times to 470 times, so the endoscopic blood loss in PELD can be calculated out by HCT-M according to the results of the in vitro experiment. Compared with the EMP-M, HCT-M is more sensitive, more accurate, and more reliable. Besides, the clinical research also found that the amount of endoscopic blood loss affects the operative procedure in operation time and fluoroscopy frequency, as well as clinical effects in VAS and ODI scores after operation in the short term.

To understand the causes of bleeding and grasp, the hemostatic measures are necessary skills for the operation of PELD. The main causes of intraoperative bleeding were analyzed as follows: (1) surgical operation injured the internal and external blood vessels of the spinal canal; (2) patients’ coagulation function was decreased; (3) anatomical factors of vertebral vein system: the venous plexus in the spinal canal, which is wrapping around the dural sac, is divided into the anterior plexus and the posterior plexus; (4) the intervertebral foramen vein is always accompanied by the nerve root, and the venous plexus in the spinal canal is connected with the celiac venous plexus [21]; and (5) surgeon’s experience is also an important factor according to the learning curve of PELD [22, 23].

**Fig. 4:**
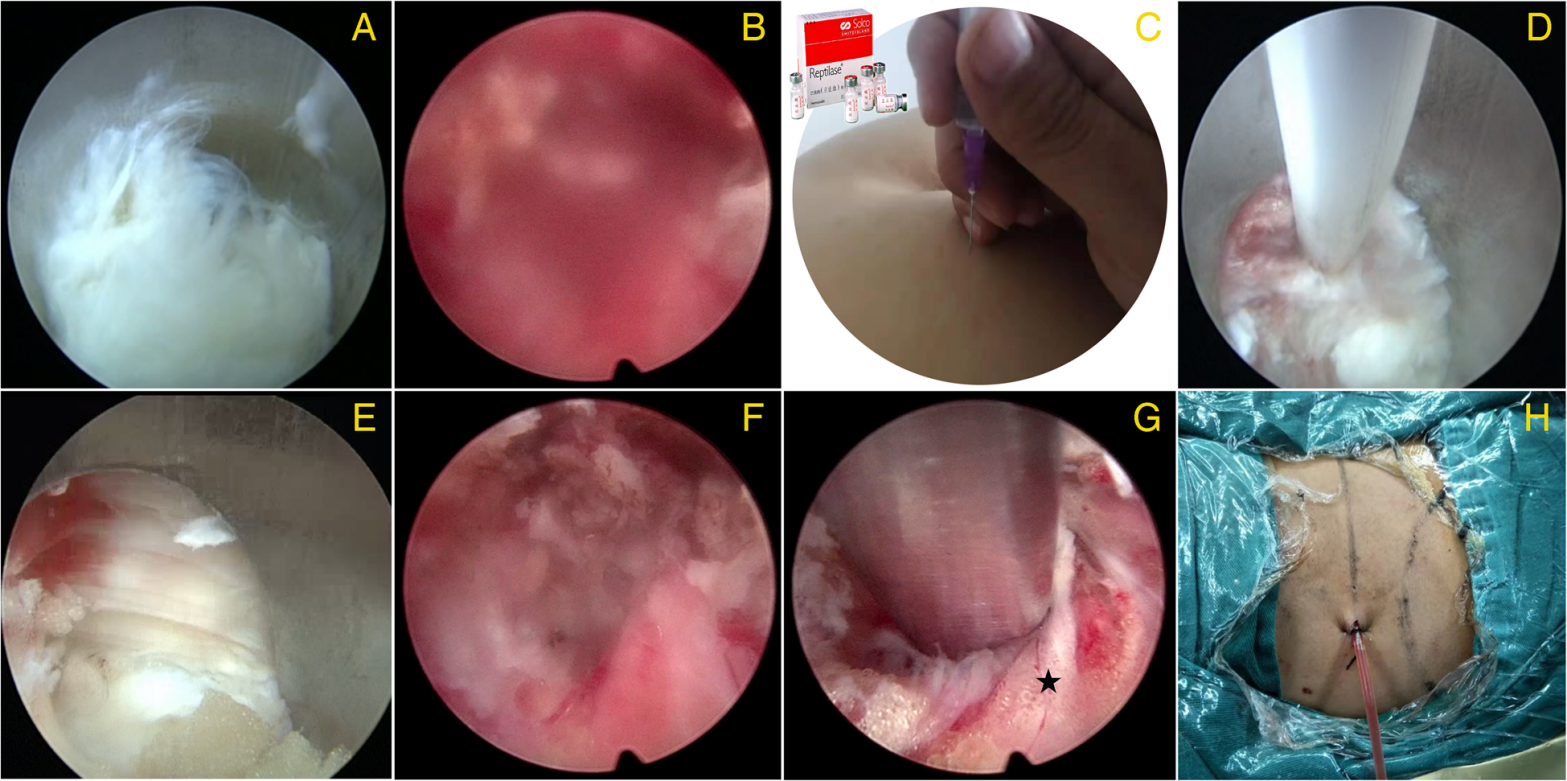
Methods to stop endoscopic bleeding. **a** The clear view under the endoscope. **b** The blood obscuring the view. **c** Intramuscular injection with hemostatic agents before operation. **d** Bipolar RF heat coagulation. **e** Working cannula compresses the bleeding point. **f** Increase the pressure of water in the spinal canal to stop bleeding. **g** Cover the bleeding area with a gelatin sponge (black star). **h** Place a drainage tube

There are several measures to stop bleeding under the endoscope (Fig. 4). First, satisfactory hemostatic effects can be obtained with hemostatic agents preoperatively or intraoperatively, such as batroxobin, tranexamic acid, reptilase, and aprotinin [24–26]. Second, taking advantage of bipolar radiofrequency (RF) heat coagulation is also a useful way to stop bleeding [27, 28]. Venous plexus hemorrhage in the spinal canal and intervertebral foramen can be stopped by bipolar RF cautery. A collapsible Elliquen bipolar RF scalpel was used during PELD, and this tool can stop the bleeding of most vessels in the spinal canal, as well as remove the granulation tissue and new blood vessels in the ruptured annulus fibrosus. Hemostasis can be realized through low temperature vaporization mode and high temperature coagulation mode. In the high temperature mode, the bipolar RF scalpel must keep away from the nerve root, but sometimes deep bleeding will suddenly obscure the field of vision, leading to an inability to distinguish the bleeding point clearly. In this situation, the high temperature mode is dangerous and the low temperature mode is useless. Thus, some other measure must be taken to stop the bleeding.

Working cannula compression is another useful way to stop bleeding. By compressing the bleeding point with the working cannula and waiting for some minutes, the bleeding will stop. In the meantime, the surgeon can turn the field of vision to the non-bleeding area, removing the nucleus pulposus and other tissues in the non-bleeding area first. However, sometimes the bleeding cannot be stopped, and the surrounding bleeding will flow into the working cannula. Another method is to increase the pressure of water in the spinal canal to form an area of high pressure outside the epidural. This method can be used for a small amount of errhysis and can decrease the time to replace the instrument. However, the time of high pressure outside the epidural should not be too long, or it will lead to intracranial hypertension, and various kinds of adverse reactions will occur (e.g., dizziness, headache, nausea and vomiting, blurred vision, tinnitus, and increased blood pressure).

Apart from the aforementioned methods, filling a gelatin sponge [29, 30] or hemostatic gauze is also frequently used to stop bleeding. When other methods are unable to stop bleeding, place a gelatin sponge or hemostatic gauze in the bleeding area and gently compress it with nucleus pulposus forceps. Generally, the bleeding can be stopped in approximately 2 min. Next, take out the foreign substances to reduce the chances of adhesion and infection in the spinal canal. Last but not the least, place a drainage tube. Some patients’ coagulation function is very poor due to the long-term use of painkillers and non-steroidal anti-inflammatory drugs (NSAIDS). These drugs affect the aggregation of platelets, increasing intraoperative bleeding during and postoperative errhysis of PELD. A drainage tube must be put in a vertebral canal hemorrhage area and connected to the negative pressure drainage ball. The tube is pulled out when the drainage fluid is less than 5 ml in 24 h after the operation. There are also some other measures that can be taken to stop the bleeding such as spraying cold saline or hemostatic agent locally [31]. In summary, surgeons should use these kinds of hemostasis flexibly according to the actual situation of operation and their personal experience.

In summary, this study based on practical problems encountered during PELD, came up with two new methods to estimate endoscopic blood loss, tested the theory through an in vitro experiment, and then came back to clinical research to confirm the results, analyze the causes of bleeding, and summarize hemostatic measures. Although the study only enrolled 74 patients with L5/S1 disk herniation and the operation method were limited to transforaminal approach, but no matter which segment of LDH and whether the posterior approach or the lateral posterior approach, the method to estimate endoscopic blood loss, the cause of bleeding, and the hemostasis are the same. So, this study can be a guideline for surgeon in PELD and all the patients with LDH can benefit from the research. The study also has limitations; bipolar RF heat coagulation may destroy some RBCs in stopping the bleeding. Although the impact is small, it may affect the results of laboratory examination.

## Conclusion

The in vitro and clinical research demonstrated that HCT-M is a reliable method to estimate endoscopic blood loss in PELD. The amount of endoscopic blood loss affects the operative procedure in operation time and fluoroscopy frequency, as well as clinical effects in VAS and ODI scores after operation in short term. Hemostatic agents, bipolar RF heat coagulation, working cannula compression, increasing the pressure in the spinal canal, filling gelatin sponge or hemostatic gauze, and placing a drainage tube are six effective methods to stop bleeding during PELD.

## Data Availability

The clinical data will not be shared in order to preserve the privacy of the patients; data can be available from authors if request.

## Abbreviations

PELD: Percutaneous endoscopic lumbar discectomy
HCT: Hematocrit
HCT-M: Hematocrit method
VAS: Visual analog scale
RBC-M: Red blood cell count method
ODI: Oswestry Disability Index
EMP-M: Empirical method
LDH: Lumbar disk herniation
YESS: Yeung endoscopic spine system
CT: Computed tomography
TESSYS: Transforaminal endoscopic spine system
MRI: Magnetic resonance imaging
RF: Radiofrequency
